# A comparative study on enhanced enzymatic hydrolysis of diverse herbaceous and woody wastes by promising dilute acid and alkaline pretreatments

**DOI:** 10.1186/s40643-025-00873-w

**Published:** 2025-04-17

**Authors:** Runxuan Shi, Zehua Zhang, Jinlei Zhang, Chang Chen, Wencheng Li, Yifan Lin, Xuyuan Shi, Peijun Zhao, Teng Zhang, Qiong Yan, Xiyu Cheng

**Affiliations:** 1 College of Life Sciences and Bioengineering, School of Physical Science and Engineering, Beijing Jiaotong University, Beijing, 100044 China; 2 Beijing Regional Center of National Narcotics Laboratory, Beijing, 100164 China

**Keywords:** Dilute acid and alkaline pretreatment, Differential response, Enzymatic hydrolysis, Herbaceous and woody wastes

## Abstract

Biomass recalcitrance makes pretreatment process a key step for efficient bioconversion process. In this study, differential effects of promising acid (AP) and alkaline pretreatments (ALP) on enzymatic hydrolysis of diverse herbaceous and woody wastes were systematically investigated. Four biomass samples were separately pretreated and sugar recovery was then recorded in the subsequent hydrolysis. Results showed that both dilute AP and ALP exhibited efficacy in the removal of hemicellulose. Specifically, soybean straw AP demonstrated the highest recovery of soluble sugars at the pretreatment stage [270 mg/g raw stalk (RS)], against 71–212 mg/g RS achieved in AP and ALP of other wastes. Compared with herbaceous soybean straw, both AP and ALP of more recalcitrant woody biomass (e.g., bamboo and poplar) showed much lower enzymatic sugar yields. Among tested samples, ALP soybean straw produced stronger structure modification, morphological changes and higher delignification, which increased its availability to cellulases. As a result, the sugar yield of ALP soybean straw using 1.5% NaOH reached 787 mg/g, which is much higher than those of other tested AP & ALP biomass wastes. The present study revealed differential responses of diverse biomass wastes to AP & ALP, hence providing valuable information for the development of effective bioconversion process of these promising biomass. Looking ahead, these classic AP and ALP will be further investigated together with other potential and emerging pretreatments (e.g., green solvent pretreatments) to provide a foundation for high value utilization of biomass.

**Graphical Abstract:**

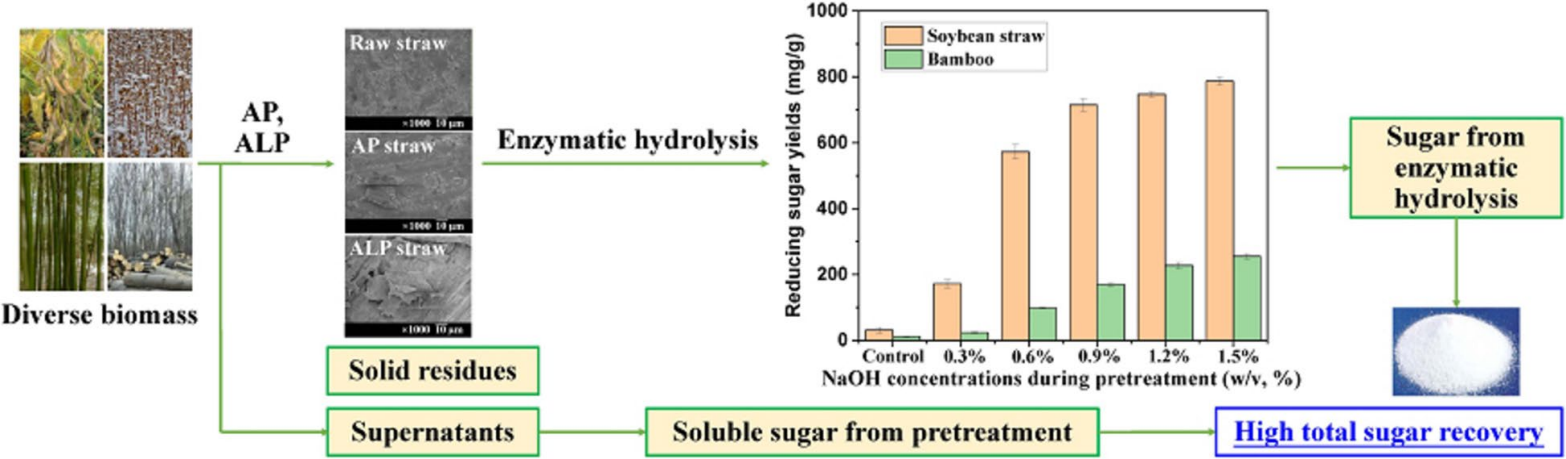

## Introduction

With the intensification of energy and environmental crises, bioconversion of different agricultural and forestry wastes has drawn considerable attention (Abd-Elhalim et al. 2015, 2019; Kang et al. 2018; Ba et al. 2024; Samantaray et al. 2024). These agricultural by-products and forestry wastes are rich in lignocellulose, and can be converted to improve its residual value. A variety of biomass substrates including cornstalk, pine foliage, cotton stalk, poplar and soybean straw have been studied for their potential to produce bio-based materials, chemicals, and biofuels, such as bioethanol or biogas (Sun et al. 2014; Lang et al. 2024; Ba et al. 2024; Wu et al. 2024). Among different biomass wastes, soybean straw and cotton straw are typical agricultural biomass, and bamboo and poplar are potential forestry wastes. The ratio of recalcitrant structure of different biomass wastes remains the main challenge of the efficiency of bioconversion (Fu et al. 2018; Sun et al. 2022; Wu et al. 2024). While the pretreatment of single biomass wastes such as corn stalk, wheat straw or some energy plant candidates (e.g. *Miscanthus*) are widely studied in bioenergy field, comparative studies on the development of promising pretreatments for these different herbaceous and woody wastes are relatively limited (Cai et al. 2024).

Many pretreatment techniques have been developed with the objective of removing lignin/hemicellulose from biomass wastes and decreasing cellulose crystallinity. These pretreatment strategies included deep eutectic solvent pretreatment (DES), liquid hot water (LHW), organic solvent, lime, oxidant, acid or alkaline solution, and ionic liquid pretreatment (Sabanci and Buyukkileci 2018; Jose et al. 2024; Wang et al. 2024; Wu et al. 2024). Efficient pretreatment processes should enhance substrate reactivity, minimize energy requirement, maximize recovery of fermentable sugars with less use of expensive chemicals (Ba et al. 2024; Samantaray et al. 2024). Until now, no individual strategy has been able to satisfy all these requirements owing to the varied physical properties of the materials.

Among different pretreatment candidates, the acid and alkaline pretreatments (i.e., AP and ALP) of lignocellulosic materials, which are relatively simple, and feasible, have great potential in large-scale biomass conversion. Lower costs and greater process reliability make AP an attractive method (Samantaray et al. 2024). Main disadvantages of acid pretreatment is its corrosion and release of by-products (Samantaray et al. 2024). The release of weak acids, furan derivatives and phenolics during the dissolution of hemicellulose during acid pretreatment has been shown to have a detrimental effect on the subsequent fermentation process, thereby reducing productivity. Similarly, ALP may produce some phenolic derivatives with complex structures. Notably, ALP is effective in lignin removal (Sabanci and Buyukkileci 2018). At the same time, corrosion problem and sugar degradation that occurred in the alkaline process are less severe than those in acid pretreatment. Some chemical pretreatment methods with severe operation did enhance the subsequent hydrolysis, however, these are associated with increased costs and the potential for the production of by-products, which can result in a reduction in efficiency during later stages (Kim 2018; Kumari and Singh 2018). Up to date, AP and ALP have improved the bioconversion of a wide range of biomass wastes including corn stalk, wheat and barley straw (Pandey and Negi 2015; Phitsuwan et al. 2016). Moreover, some studies found that AP/ALP did remove hemicellulose/lignin components, but it failed to result in a greater yield of fermentable sugars (only 91–92 mg/g), and the efficiency of AP/ALP may vary greatly in different studies (Pandey and Negi 2015; Chakraborty et al. 2024). The precise function of chemical pretreatment in enhancing the hydrolysis efficiency of diverse biomass substrates were intricate, thereby necessitating additional research to elucidate the underlying mechanisms.

This study aims to illustrate the differential response of herbaceous and woody plants to these relatively simple, feasible and promising chemical pretreatments (i.e., AP and ALP) through a systematic comparative study. In brief, the effect of AP and ALP with mild operations on enzymatic hydrolysis of diverse herbaceous and woody wastes (i.e., soybean straw, cotton straw, bamboo and poplar wastes) was methodically investigated in order to establish a cost-effective pretreatment strategy for enhancing processing efficiency. The composition and microstructure of the substrates were recorded to facilitate a more profound understanding of the responses of varied biomass wastes to each pretreatment. This was achieved with a view to understand how biomass recalcitrance was altered, and how this can result in improvements to subsequent enzymatic hydrolysis. A further investigation was undertaken into the fermentable sugar recovery, for the purpose of evaluating their potential for application in biofuel production.

## Materials and methods

### Materials

Soybean straw, cotton straw, bamboo and poplar wastes were obtained from Jiangsu province, China. These biomass substrate were placed in a Thermo Fisher OMH180 oven (USA) for more than 24 h until the weight is constant. These samples were treated using a plant miller (40 mesh).

### Pretreatment process

Mild operational conditions were chosen for pretreatment in this work for less corrosion problems based on previous studies (Zhang et al. 2018; Cheng et al. 2022). Biomass substrates were treated using 1.0%–4.0% H_2_SO_4_ (w/v) (AP) or 0.3%–1.5% NaOH (w/v) (ALP) solutions (solid rate 1:10) in an autoclave (121 °C, 30 min). Following centrifugation (Thermo Fisher LEGEND MICRO 21R, USA), the obtained liquid samples were stored for subsequent experiments. The remained residues were collected and washed until the pH reaches around 7. Subsequently, they were placed in a Thermo Fisher OMH180 oven (USA) (105 °C) until the weight is constant. The dried solid residue is collected for the subsequent experiments.

### Enzymatic hydrolysis

Hydrolysis of biomass wastes was conducted with an enzyme system (15 FPU/g of the substrates) consisting of 2.5% solid loading, 40 μL tetracycline hydrochloride and 50 mM buffer (pH 5). A shaker was used to mix the solutions at 150 rpm and 50 °C for 72 h (shaker, Shanghai Zhicheng Instrument ZWY-240, China). The above enzyme was bought from Utter Biochemical of China. The supernatant was then collected as much as possible. The residue samples were collected using centrifugation. Reducing sugar were tested using previous procedure (Miller 1959).

#### X-ray diffraction (XRD), Fourier transform infrared spectra (FTIR) and scanning electron microscopy (SEM) observation

Biomass samples were observed using a FTIR spectrometer (Shimadzu IRTracer-100, Japan). XRD analysis was conducted using the previous Segal peak height method, and we calculated the crystallinity index (CrI) by using the following equation (I_am_, the minimal diffraction intensity of amorphous region; I_002_, the maximum diffraction intensity) (Xing et al. 2018; Salem et al. 2023):

1 $${\text{CrI}}\left( \% \right) = {1}00 \times \left( {{\text{I}}_{{00{2}}} - {\text{I}}_{{{\text{am}}}} } \right)/{\text{I}}_{{00{2}}}$$

The microstructural changes of biomass samples before and after different pretreatments were observed by a JEOL JSM-5600 LV SEM (Tokyo, Japan) (1000×).

### Analytical methods

Samples were collected following a series of pretreatments and subsequent centrifugation. The resulting clear upper layer can be utilized for the analysis of soluble sugars. The remaining solid samples were subjected to a drying process. The results of total solids and soluble sugars were recorded as before (Miller 1959; APHA 1998). The contents of lignocellulosic components of different materials were carried out using previous procedures (Goering and Van-Soest 1970).

The conversion ratio (CR) was measured using the following method (Phitsuwan et al. 2016; Tang et al. 2019):

2 $${\text{CR}}\left( \% \right) = {1}00 \times {\text{YRS}}/\left( {{1}.{111} \times {\text{H}}_{{\text{C}}} + {1}.{136} \times {\text{H}}_{{{\text{HC}}}} } \right)$$

In this study, YRS (g/g substrate) denoted the yield (reducing sugar) per gram of substrate; H_C_ is the cellulose content (g/g substrate); H_HC_ is the hemicellulose content (g/g substrate); the constant 1.111 is a factor for the equivalent sugar amounts from per gram of cellulose; the constant 1.136 is a factor for the equivalent sugar amounts from per gram of hemicellulose.

The data in this work were analyzed using IBM’s SPSS 2 software and statistical significance analysis was carried out by the Duncan method (*p* < 0.01). We did all of experiments in triplicate. The results are the average values of triplicate experiments plus the standard deviation.

## Results and discussion

### Effect of AP on the dissolution of components of diverse herbaceous and woody wastes

**Fig. 1:**
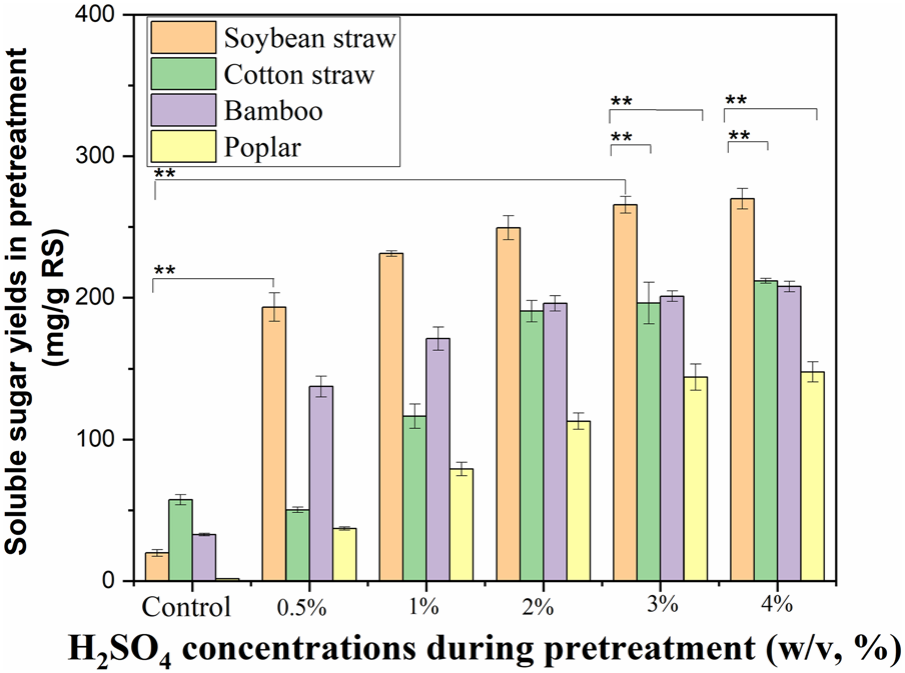
Effect of AP on the soluble sugar yields in the pretreatment stage. **A** Soybean straw and cotton straw; **B** bamboo and poplar. The soluble sugar yield in the pretreatment process was calculated based on per g raw stalk (RS). Values are means of triplicate ± standard deviation. AP conditions: 0.5%-4% H_2_SO_4_ solution, 121 °C for 30 min

It is evident that diverse pretreatments can effectively remove lignin/hemicellulose and mitigate recalcitrance levels of lignocellulosic biomass, thereby enhancing accessibility of cellulose to the cellulase (Brienzo et al. 2017). AP was used to pretreat the selected herbaceous and woody wastes and decrease the recalcitrance of the biomass. The solid yields and dissolution of components in different pretreatments are recorded (Table 1 and Fig. 1). Results show that lignocellulosic components of all tested biomass wastes were significantly dissolved during AP. The solid yields of herbaceous soybean straw were between 59.7 and 47.8% in AP (Table 1). In the cases of woody biomass wastes (cotton straw, bamboo and poplar wastes), the weight loss was also higher in AP with 4.0% H_2_SO_4_, indicating the dissolution of lignocellulosic components (e.g., hemicellulose) (Ba et al.^2024^; Chen et al.^2024^). Compared with herbaceous biomass (i.e., soybean straw), the solid recovery rate in AP of woody biomass wastes is generally higher, indicating that these woody biomass wastes are more recalcitrant than soybean straw.

**Table 1:** Effect of AP on solid yields of diverse herbaceous and woody wastes

Acid level (%, w/v)	Solid yield (%)			
	Soybean straw	Cotton straw	Bamboo	Poplar
Control	79.0 ± 0.3	81.3 ± 1.2	85.5 ± 0.3	78.5 ± 1.1
0.5	59.7 ± 1.7**	81.4 ± 0.7	73.8 ± 0.4**	73.8 ± 3.5**
1	50.8 ± 0.1**	70.6 ± 4.0**	68.8 ± 0.9**	66.3 ± 3.1**
2	48.9 ± 1.6**	62.8 ± 1.1**	66.8 ± 0.4**	62.1 ± 1.0**
3	48.6 ± 1.3**	61.7 ± 0.4**	65.9 ± 0.4**	61.5 ± 1.7**
4	47.8 ± 0.1**	58.9 ± 0.8**	64.8 ± 0.4**	59.1 ± 0.4**

^**^ *p* < 0.01

The reduction in the mass of biomass waste can be explained by the dissolution of its components into water-based solutions. (Fig. 1). The soluble sugar after AP with 0.5% H_2_SO_4_ reached 193 mg/g raw stalk (RS) of soybean straw, against 20 mg/g RS in the control (Fig. 1). The lower solid yield (or higher weight loss) in AP with higher 4% H_2_SO_4_ concentrations was recorded. As a result, the soluble sugars were higher (270 mg/g RS) in the case of soybean straw. When the concentration of dilute H_2_SO_4_ was increased to a certain extent, the increase in soluble sugar production during the pretreatment process became insignificant.

In the cases of cotton straw, the soluble sugar yields also increased with the improved H_2_SO_4_ concentrations in AP. In comparison to soybean straw, the significantly higher solid yields in AP of bamboo and poplar were observed (*p* < 0.01), and AP of these biomass wastes thereby gave much lower soluble sugar levels (137–208 and 37–148 mg/g RS vs. 193–270 mg/g RS). It can be attributed to the higher recalcitrance of cellulose in bamboo and poplar, which renders them more resistant to degradation in AP (Kassaye et al. 2017).

### Effect of AP on sugar recovery of diverse wastes in enzymatic hydrolysis

**Fig. 2:**
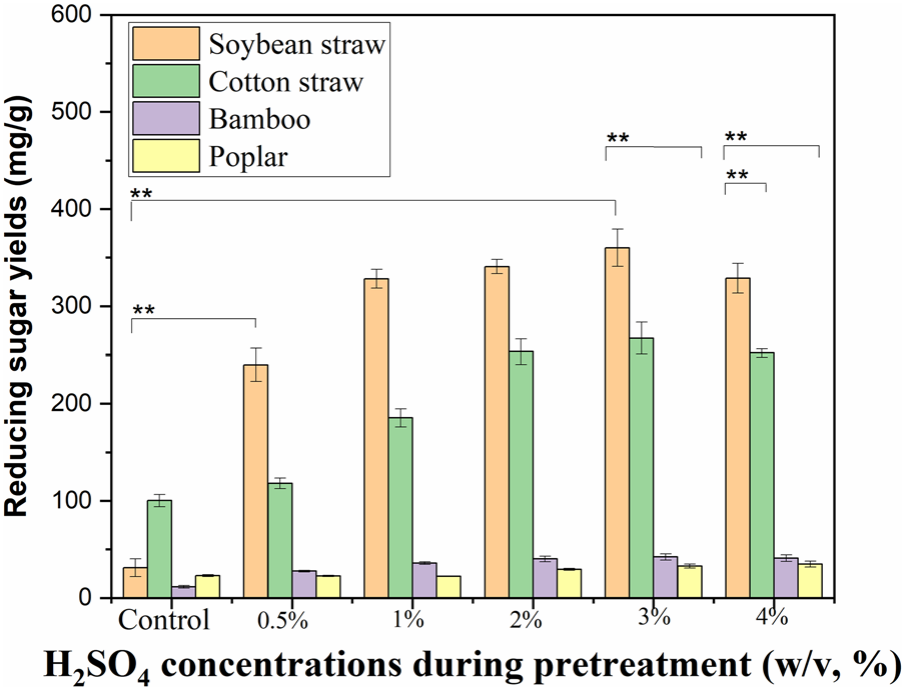
Effect of AP on enzymatic hydrolysis of diverse herbaceous and woody wastes. **A** soybean straw and cotton straw; **B** bamboo and poplar. AP conditions: 0.5%-4% H_2_SO_4_ solution, 121 °C for 30 min

Subsequently, the biodegradability of the different biomass wastes that have been pretreated with AP was assessed by means of enzymatic hydrolysis. Sugar yield of the untreated soybean straw sample was only 31 mg/g, and it became higher in the case of AP with 0.5% H_2_SO_4_. When H_2_SO_4_ concentration was improved, the enzymatic sugar yield of soybean straw significantly increased (*p* < 0.01). At 3% H_2_SO_4_ solution, the sugar yield of soybean straw was 360 mg/g, which is about 11-fold higher than that of the control. At higher H_2_SO_4_ concentration, the sugar yield showed a slight decrease (Fig. 2).

Similarly, the enzymatic sugar production of cotton straw was remarkably enhanced in AP (*p* < 0.01). Reducing sugar recovery of pretreated cotton straw by 0.5% H_2_SO_4_ solutions reached 118 mg/g. With the increase of H_2_SO_4_ concentration, the enzymatic sugar yield of pretreated cotton wastes also significantly increased, and the highest level of 267 mg/g was observed in AP with 3% H_2_SO_4_ solutions.

The microstructures of the lignocellulosic samples also rendered the enzymatic saccharification process (Chen et al. 2024). Compared with soybean and cotton straw, the corresponding enzymatic sugar yields of bamboo and poplar substrates pretreated by AP is much lower. The reducing sugar yields of bamboo and poplar wastes pretreated by 0.5% H_2_SO_4_ solutions were only 28 and 23 mg/g, respectively. With the increase of H_2_SO_4_ concentration, the enzymatic sugar yields of woody biomass wastes showed a slight increase. At 3–4% H_2_SO_4_ solution, the corresponding sugar yields of bamboo and poplar wastes were only 42 and 35 mg/g, respectively. The limited enhancement effect of enzymatic hydrolysis should be closely related to the recalcitrant characteristics of bamboo and poplar. Achieving optimal enzymatic sugar yields from recalcitrant woody raw materials, such as bamboo and poplar, poses a significant challenge. Consequently, there is an imperative to identify more efficacious pretreatment methodologies (Chakraborty et al. 2024).

### Effect of AP on composition of diverse biomass wastes

The efficacy of acid pretreatment can be evaluated by the solid yield and changes in sample composition. The removal of the hemicellulose component of the sample, which is the primary cause of the alteration in sample mass after AP (Cheng et al. 2022). The dry matter of diverse herbaceous and woody biomass wastes retained after different pretreatment was about 47.8–81.4% (Table 2). The observed weight loss may be attributed to the extraction of hemicellulose from the biomass samples. The hemicellulose levels decreased to 5.83% in the case of AP soybean straw samples, and the corresponding cellulose content thereby exhibited a considerable increase from 33.3 to 55.8% due to hemicellulose removal. In AP of different woody wastes, hemicellulose removal was also observed and the corresponding cellulose contents increased to 31.7%–59.2%. In comparison to soybean straw, significantly lower hemicellulose removal and weight loss were observed in the cases of AP of bamboo and poplar wastes (Tables 1 and 2).

**Table 2:** Effect of AP on chemical composition of diverse herbaceous and woody wastes

Samples	Cellulose content (%)	Hemicellulose content (%)
Raw soybean straw	33.3 ± 0.1	32.5 ± 1.2
AP soybean straw	55.8 ± 1.2**	5.83 ± 1.2**
Raw cotton straw	21.7 ± 2.3	27.5 ± 1.2
AP cotton straw	31.7 ± 0.1**	7.50 ± 3.5**
Raw bamboo	34.2 ± 1.2	23.3 ± 2.4
AP bamboo	47.5 ± 1.2**	4.17 ± 1.2**
Raw poplar	47.5 ± 1.2	22.5 ± 1.2
AP poplar	59.2 ± 1.2**	5.83 ± 1.2**

AP was carried out with 3% H_2_SO_4_

Values are means of triplicate ± standard deviation. **, *p* < 0.01

As a result, AP of these woody wastes indicated much lower lignocellulosic removal and sugar release (Fig. 1). When compared with cellulose, the elimination of hemicellulose was a comparatively straightforward process (Samantaray et al. 2024). Pretreatment with dilute acid has been shown to randomly open the glycosidic bonds of the biomass material and remove hemicellulose, thus increasing the corresponding cellulose level (Shimizu et al. 2018). Therefore, the accessibility of the diverse biomass substrates is significantly improved after AP.

### Effect of AP on microstructure of diverse biomass wastes

#### SEM observation

**Fig. 3:**
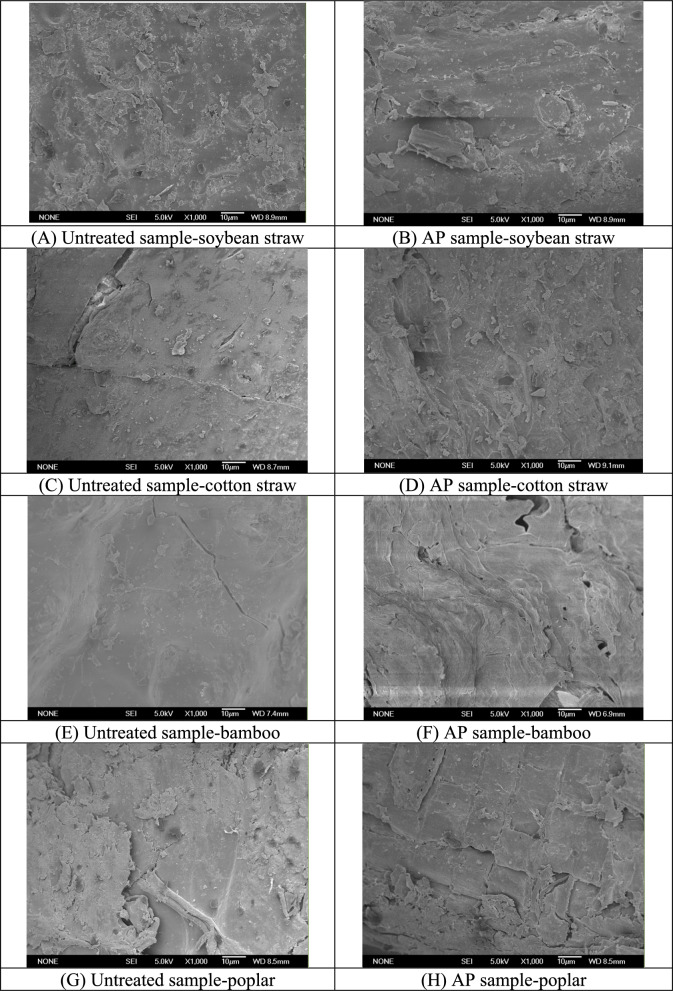
SEM images of biomass samples with and without pretreatments (1000×)

The alterations in the microstructure of biomass samples after AP pretreatment were likewise examined (Fig. 3). Raw soybean straw exhibited a smooth surface and maintained its fibrous microstructural arrangement (Fig. 3A). In contrast, the cell walls of the soybean straw in the AP sample underwent significant modifications. As Fig. 3B shows, it is obvious that the surfaces of cell were not smooth but partly broken, and the formation of cracks and fragments were observed, thereby enhancing the accessibility of stalk sample to cellulases.

The microstructure changes of woody biomass samples after AP pretreatment were also observed by SEM at the selected magnification (1000×, Fig. 3C–H). Raw cotton straw and bamboo samples also had a smooth surface (Fig. 3C). The cell walls of the cotton straw sample were changed after AP, and the cell surface became uneven (Fig. 3D). Strong morphological modifications were evident in the AP sample of bamboo (Fig. 3E and F). The surface of the cell walls appeared twisted, and several pores were discernible in AP of the bamboo. It was observed that the AP poplar sample was likewise not smooth but partially fragmented. Additionally, some fragments were observed from the pretreated sample. Significant changes of microstructure were reported in other chemcial pretreatments of biomass materials (Liu and Cheng 2010). These results in this section suggested that AP led to a substantial disruption in the microstructure of the cell walls. The induction of cracks, fragments, and cell structural distortion by AP can make them accessible, thereby accelerating their bioconversion.

#### XRD analysis

**Fig. 4:**
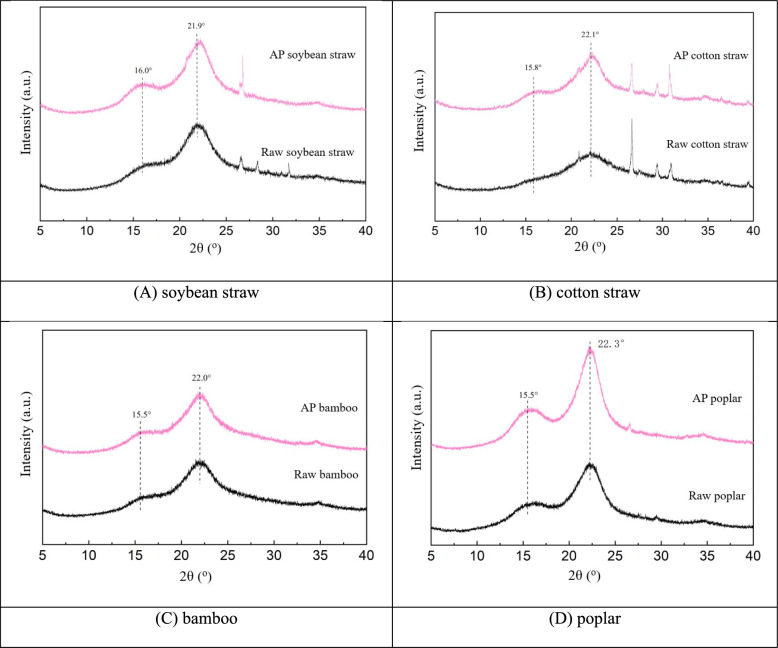
XRD analysis of biomass samples with and without pretreatments

In addition to changes in microstructure, crystallinity is also a crucial factor to consider when assessing biomass cellulose hydrolysis rates (Sandy et al. 2010). XRD is a simple, widely used and powerful non-invasive analytical technique used to characterize cellulosic materials, providing information on structural parameters such as crystal orientation and crystallinity (Xing et al. 2018; Salem et al. 2023). Therefore, the crystal structure of diverse herbaceous and woody biomass wastes recorded using XRD analysis. These samples exhibited three distinct peaks at 2θ = 16°, 22° and 35° simultaneously, which are characteristic of the various crystallographic planes associated with crystalline cellulose I (Fig. 4) (Wang et al. 2018). The peak height reflects the quantity of crystalline regions, and higher peak heights indicate higher crystallinity. And peak width reflects crystal size, narrow peaks suggest large crystal sizes while wide peaks reveal small crystal sizes (Salem et al. 2023). The increase in the intensity of the diffraction peaks after acid treatment showed that AP changed the morphology of the components of biomass and the compactness of the cellulose by removing the amorphous components in the straw, which is conducive to promoting the following enzymatic hydrolysis process. In addition, the intensity of the scattered peaks of cotton and soybean straws was greatly weakened after acid pretreatment, indicating that the acid pretreatment removed some of the impurities or non-cellulose and other substances in the straws, reduced the scattering of X-rays by these components, and indirectly facilitated the appearance of the solid cellulose crystalline form of the straws. Narrow peak shapes can be seen after pretreatment in poplar, suggesting that the acid treatment may have increased the crystallinity or crystal size of poplar or optimized the crystal structure, thereby resulting in narrower diffraction peaks (Wang et al. 2018).

The CrI represents the relative content between the crystalline and amorphous zone. This parameter can roughly indicate the cellulose content in the biomass using a two-phase model (crystalline and amorphous phases), which is an important index of the accessibility in the following bioconversion of lignocellulosic substrates (Wang et al. 2018). The CrI was assessed using the Segal's crystallinity index approach, which is well-suited for studying the relative change in crystallinity due to the exposure of samples to different treatments (Park et al.^2010^; Xing et al. 2018; Salem et al. 2023). After subtracting the diffraction intensities for non-crystalline background diffraction, CrI can be derived from the proportion of the crystalline peak intensity (I_002_–I_AM_) relative to the overall intensity (I_002_) (Xing et al. 2018; Salem et al. 2023). In comparison to raw samples, all pretreated samples indicate higher CrI values, elevation in the crystallinity index resulted in enhanced accessibility of these pretreated substrates (Fig. 4). Acid hydrolysis produced a decrease of polymerization degree, and it reduced the constraints on the molecular motions of the cellulose, allowing them to adopt a more ordered arrangement, leading to an increase in crystallinity, consistent with previous research reports (Sandy et al. 2010). As shown in Table 2, AP significantly removed hemicellulose, and thereby gave higher cellulose content in the remained biomass, which is corresponding well with the higher crystallinity. Increased CrI value of the pretreated biomass is due to the efficient elimination of lignocellulosic components during the pretreatment process. Undoubtedly, this improvement is advantageous for the following bioconversion (Wang et al. 2018). Moreover, the cellulose in the amorphous zone and the surface of the crystalline zone could rearrange itself during drying at high temperature and form intermolecular hydrogen bonds, which may also be one of the reasons for the increase in crystallinity (Toba et al. 2013).

#### FTIR analysis

**Fig. 5:**
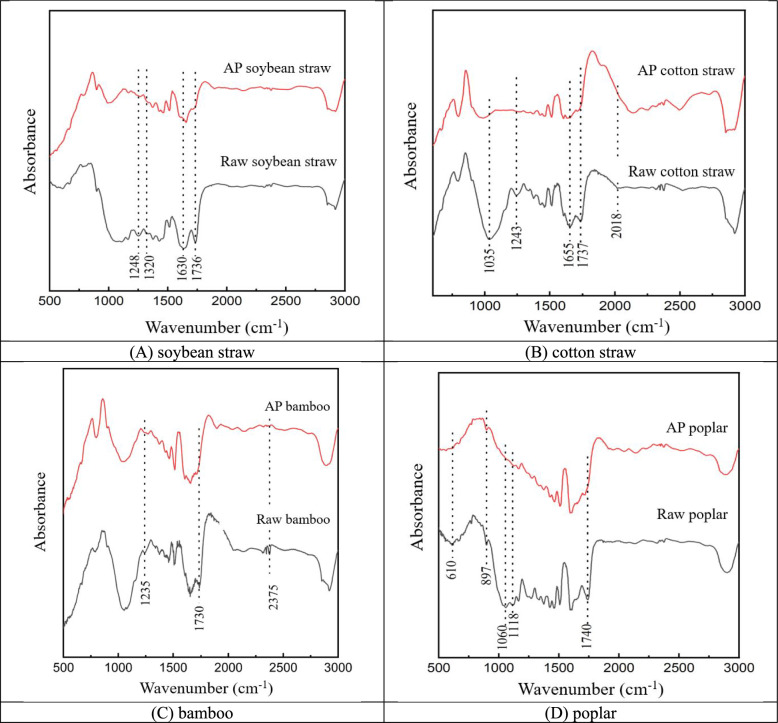
FTIR analysis of biomass samples with and without pretreatments

Alterations in functional groups and chemical characteristics exert a crucial influence on the enzymatic hydrolysis process, and the corresponding structural changes happening during pretreatment can be observed by FTIR analysis (Fig. 5).

The carbonyl groups (C=O) absorption peak related to the side chains of hemicellulose or lignin corresponds to a position around 1740 cm^−1^. Results indicated that AP decreased this peak intensity in four pretreated wastes while it is prominent in four untreated biomass samples, confirming the change in lignin and the disruption of its connection with hemicellulose. The level of C=O peak (1740 cm^−1^) is positively correlated to hemicellulose content. It could be resulted from the break of hemicellulose ester bands in pretreatments (Wang et al. 2018). In deconstruction process of different biomass wastes, acetyl groups in lignocellulosic components (e.g., hemicellulose) play a vital role. And deacetylation reaction in hemicellulose could help to significantly increase the bioconversion (Hsu et al. 2009). Obvious decreases of absorption peak intensity ranging from 1035 to 1380 cm^−1^ (e.g., stretching of C–O and C=O, 1035 cm^−1^; distortion of C–H, 1375 cm^−1^) (Chakraborty et al. 2024) in four pretreated biomass samples were also observed, which is corresponding with the loss of hemicellulose (Table 2 and Fig. 4). The C–O–C bond is the main molecular connection in lignocellusic components (lignin/hemicellulose). These changes indicate a certain degree of dissociation of lignocellusic components (Wang et al. 2018). In FTIR, the characteristic vibration peak of β-(1,4) glycosidic bond at wavenumber 897 cm^−1^ (Wang et al. 2018) indicated slight enhancement for the pretreated materials. It suggests that the cellulose content in samples pretreated with AP is greater than in the untreated ones, as revealed by the composition changes (Table 2). All these changes are beneficial for the following enzymatic hydrolysis process and further recycle of fermentable sugars (Fig. 2).

In lignocellulosic wastes, the recalcitrant crystalline structure and the barrier of lignin significantly impede the bioconversion (Jin et al. 2016). The dramatical increase of reducing sugar yields was due to the reduction of hemicellulose content and variations in cell microstructure appearing after AP treatment (Figs. 3, 4, 5). The soybean straw pretreated with AP, which had a greater amount of hemicellulose removed, exhibited a significantly higher reducing sugar yield than the pretreated bamboo and poplar samples. This is because the removal of hemicellulose by AP increases the cellulose substrate content, thereby enhancing the bioconversion (Jin et al. 2016; Wang et al. 2018), which is crucial for improving enzymatic hydrolysis. Compared with bamboo and poplar substrates, stronger disruption of cell structure and greater enhancement of cellulose accessibility in the pretreated soybean straw were confirmed by the corresponding SEM observations (Fig. 3), which also had positive effects on enzymatic hydrolysis. Compared with soybean and cotton straw, the reducing sugar yields of AP pretreated bamboo and poplar are much lower (35–42 vs. 267–360 mg/g), which could be attributed to the limited structure modifications during AP of these recalcitrant woody biomass (Table 2, Figs. 3, 4, 5) (Chen et al. 2024).

### Effect of ALP on enzymatic hydrolysis of diverse wastes

#### Effect of ALP on dissolution of components

Moreover, ALP was further investigated to improve the bioconversion of the representative of woody and herbaceous plants (i.e., soybean and bamboo wastes). Lignocellulosic components of the above biomass wastes were significantly dissolved during ALP, and with the increase of NaOH pretreatment concentration, a concomitant decrease in solids recovery can be observed in ALP. The solid yields of soybean straw and bamboo in ALP with alkali addition of 0.3% to 1.5% were 75.9–45.8% and 88.5–58.8% respectively, indicating the higher recalcitrance of bamboo biomass than that of soybean biomass. The recovery of solids following ALP has been shown to be comparable to that achieved through AP. Therefore, the corresponding highest soluble sugar levels were recorded in the aqueous solutions of soybean straw and bamboo wastes pretreated by ALP with 1.5% NaOH, respectively. In comparison to AP, ALP of these biomass wastes gave lower soluble sugar levels (Tables 1 and 3), indicating that AP is more effective than ALP in dissolving the hemicellulose components into the soluble sugars. It could be due to the conversion of these sugars into other by-products following dissolution (Cheng et al. 2022).

**Table 3:** Effect of ALP on solid yields and dissolution of components of diverse herbaceous and woody wastes

Alkali level (%, w/v)	Solid yield (%)		Soluble sugar yield (mg/g RS)^a^	
	Soybean straw	Bamboo	Soybean straw	Bamboo
Control	79.0 ± 0.3	88.5 ± 3.1	19.8 ± 2.4	32.7 ± 1.0
0.3	75.9 ± 0.4	81.7 ± 0.4**	32.9 ± 6.2**	23.5 ± 0.6**
0.6	63.9 ± 0.1**	75.3 ± 0.4**	86.7 ± 5.0**	40.5 ± 1.5**
0.9	55.2 ± 0.3**	69.0 ± 0.3**	110.4 ± 5.0**	50.5 ± 0.8**
1.2	50.1 ± 0.3**	63.3 ± 0.4**	123.1 ± 4.3**	65.6 ± 2.6**
1.5	48.8 ± 0.2**	58.8 ± 0.2**	134.0 ± 7.1**	70.5 ± 3.5**

^a^The soluble sugar yield in the pretreatment process was calculated based on per g raw stalk (RS). Values are means of triplicate ± standard deviation. **, *p* < 0.01

#### Effect of ALP on enzymatic hydrolysis

**Fig. 6:**
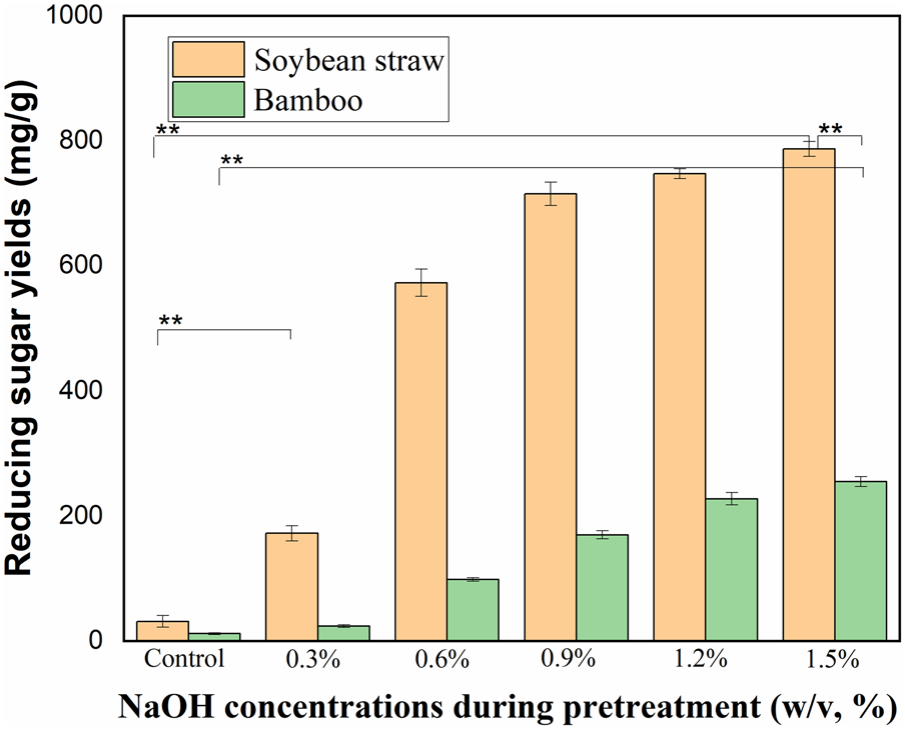
Effect of ALP on enzymatic hydrolysis of diverse herbaceous and woody wastes. ALP conditions: 0.3%–1.5% NaOH solution, 121 °C for 30 min

The bioconversion efficiency of different biomass wastes pretreated by ALP was then studied. As shown in Fig. 6, ALP catalyzed by different concentrations of NaOH of soybean straw were effective in promoting the subsequent enzymatic hydrolysis, and the enzymatic sugar level increased gradually with the increase of NaOH pretreatment concentration. Samples pretreated with 0.3% NaOH solution under 121 °C for 30 min showed a significant improvement of sugar recovery (*p* < 0.01). With the increase of NaOH concentration, the enzymatic sugar yield of soybean straw significantly increased. At 1.5% NaOH solution, the sugar yield and conversion ratio of soybean straw reached the highest level of 787 mg/g and 92.1%, respectively. The enzymatic sugar production of bamboo was also significantly enhanced by ALP (*p* < 0.01). Overall, enzymatic sugar yields from ALP substrates showed an increasing trend. The sugar recovery reached 255 mg/g in ALP bamboo pretreated by 1.5% NaOH solution, which is much higher than those obtained from raw bamboo as well as AP bamboo samples (42 mg/g). However, the sugar recovery levels were still significantly lower than that of ALP soybean straw. The existence of different responses to different pretreatment processes for different woody plant feedstocks was also a further justification for the necessity of studies into differential biomass pretreatment (Chen et al. 2024).

#### Effect of ALP on composition and microstructure

**Fig. 7:**
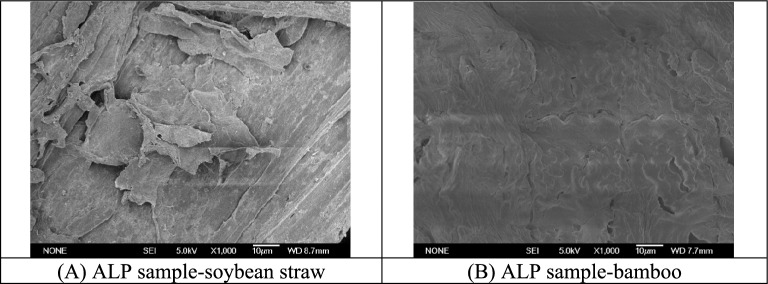
SEM images of biomass samples with ALP (1000×)

Microstructure changes in ALP were also observed by SEM at the selected magnification (Fig. 7). After ALP, the cell walls of bamboo were damaged to some extent, and distorted structures were observed on the surface. Improved degradation of the cell wall has likewise been documented in previous research involving microwave-assisted chemical pretreatments of lignocellulose (Jin et al. 2016). Compared with bamboo, ALP produced much stronger structural damage to soybean straw. As depicted in Fig. 7, surfaces and cell walls of ALP-treated soybean straw sample underwent substantial disruption and deformation, with a multitude of fissures and fragments emerging on the surfaces. These alterations are likely to generate numerous reactive sites on surface of the sample, thereby enhancing the accessibility of the straw sample to enzymatic action and, in turn, expediting the following bioconversion process. As a result, soybean straw pretreated by ALP with 1.5% NaOH achieve the best sugar recovery.

### Comparison of the effects of chemical pretreatments on enzymatic hydrolysis

Enzymatic hydrolysis was severely affected by the lignin barrier and resilient network in lignocellulose formed by cellulose, hemicellulose and lignin (Ba et al.^2024^; Chen et al. 2024). AP is effective in removing the hemicellulose and opening cellulose-hemicellulose-lignin network, hence indicating enhanced sugar recovery. The pretreated sample by ALP, which is much more effective on delignification, exhibited a significantly higher yield of reducing sugars compared to the AP samples. These findings strengthen the viewpoint that it is important to remove lignin for improving bioconversion efficiency. The delignification notably diminished the cellulase adsorption onto lignin, and increased degradable cellulose content, thereby making pretreated samples more accessible to cellulase (Ba et al. 2024; Chen et al. 2024). Stronger cell wall destruction of ALP samples affirmed by corresponding SEM observation and higher accessibility definitely benefited the subsequent hydrolysis.

It is well known that some toxic byproducts (e.g., organic acids) may be generated by some pretreatments (e.g. some chemical treatments with a high temperature at 160–260 °C) (Ximenes et al. 2011; Ba et al. 2024;). Current AP and ALP of soybean and cotton straw remained effective, as evidenced by the high sugar yields in the following hydrolysis (see Figs. 2 and 6). This suggests that cellulase activity was not significantly hindered during the process. These findings further validate the efficacy of these chemical pretreatment approaches for agricultural biomass conversion. The minimal inhibitory effects observed can be primarily ascribed to the relatively mild operating conditions employed (low alkaline/acid concentrations, 121 °C), which could lead to lower levels of by-products (Chen et al. 2024).

In the future ethanol or biogas fermentation process using the corresponding pretreatment liquid and pretreated biomass as the substrates, treatment based on acid–base neutralization reaction could be used (Prasad et al. 2018; Yu et al. 2019). The adsorbents like activated charcoal or the incorporation of proteins/surfactants may serve to eliminate the possible inhibitory effect of the by-products on the microorganisms (Prasad et al. 2018; Sun et al. 2022), thereby enhancing the bioconversion process. Furthermore, some mature processes can be used for large-scale recycling and utilization of acid–base waste liquids. For example, black liquor produced in the alkaline pretreatment can achieve the recycling of alkali through a series of steps such as extraction, evaporation, combustion, causticization, and lime recovery (Chakraborty et al. 2024).

An effective pretreatment strategy should not only facilitate the production of easily hydrolyzable substrates but also optimize the overall recovery of fermentable sugars (Cui and Bai 2024). Under the optimal AP conditions for soybean straw, the maximum reducing sugar concentration in the aqueous phase reached 270 mg/g RS, while the corresponding yield from enzymatic hydrolysis achieved 360 mg/g. The sugar recovery in ALP increased to 787 mg/g and 255 mg/g in the case of soybean straw and bamboo samples, respectively. The highest sugar release, ranging from 147 to 629 mg/g, was achieved by AP/ALP of various wastes (e.g., bamboo, stalk, and straw) (Eliana et al. 2014; Obeng et al. 2018; Wang et al. 2018). The fermentable sugar recovery (360 and 787 mg/g pretreated stalk) and conversion ratios (50.8% and 92.1%) from soybean straw sample pretreated by AP and ALP was also comparable to previous results (Table 4). Compared with soybean straw, both AP and ALP were not efficient enough to enhance the bioconversion of bamboo, and further research is underway to develop efficient pretreatment methods for the recalcitrant woody biomass waste.

**Table 4:** Comparison of fermentable sugar recovery from different biomass

Biomass	Pretreatment conditions	Sugar yield (mg/g)^a^	Conversion ratio (%)^b^	Refs.
Bamboo	Tween 80 with 1% H_2_SO_4_; 121 °C for 60 min	153	24.7	Obeng et al. (2018)
Pine foliage	C-TAB with 1% H_2_SO_4_; PEG with 1% NaOH; 121 °C for 60 min	477–588	88.4–98.1	Pandey and Negi (2015)
*Pennisetum purpureum*	1.5% NaOH, 121 °C for 60 min	147	–	Tsai et al. (2018)
*Pennisetum purpureum*	2% Ca(OH)_2_/NaOH, 121 °C for 60 min	324–537	65.5–88.7	Phitsuwan et al. (2016)
Soybean and cotton straw	3% H_2_SO_4_, 121 °C for 30 min	267–360	50.8–61.0	This work
Soybean	1.5% NaOH, 121 °C for 30 min	787	92.1	This work
bamboo	1.5% NaOH, 121 °C for 30 min	255	29.0	This work

^a^The yield were obtained from per g pretreated substrate

^b^The conversion ratio is calculated using the standard method described in the analytical methods of “Materials and methods” section

In the present work, the differential response of different herbaceous and woody plants to these classic and promising chemical pretreatments have been revealed through a systematic comparative study. In addition to the classic, and promising AP and ALP explored in this study, steam explosion is one of ecofriendly, and inexpensive methods (Hoang et al. 2023). The application of DES still has a long way to go, but it does indicate potential as an efficient solvent (Wang and Lee 2023). Recently, consideration for environmentally benign and sustainable options, such as solvents derived from biomass has attracted increasing attention. Solvents derived from carbohydrates and lignin have been proposed and evaluated as eco-friendly options in numerous biomass conversion processes (Meng et al. 2023). Further studies will be carried out to develop novel pretreatment strategies of these classic, potential, emerging pretreatments and/or their combinations, hence making bioconversion of these materials become a valuable and promising avenue for bioenergy production.

## Conclusions

The transformation of agricultural and forestry wastes into valuable commodities is a strategy that offers both economic gain and carbon emission reduction. Our study comparatively analyzed the response of various herbaceous and woody plants to AP and ALP pretreatments, revealing distinct mechanisms. AP and ALP induced structural changes and component removal in biomass wastes. Notably, the highest level (787 mg/g) of reducing sugar was observed in ALP with 1.5% NaOH. In contrast, woody biomass treated with AP and ALP produced lower enzymatic sugar yields. Among tested cases, ALP soybean straw exhibited stronger structure modification, morphological changes and higher delignification, which could significantly increase the accessibility of the pretreated wastes to enzymes, and consequently generate a significantly higher reducing sugar yield compared with other tested AP and ALP biomass wastes. These findings would enhance our understanding of biomass-enzyme interactions during enzymatic hydrolysis. In conclusion, our study highlights the differential effects of classic AP and ALP pretreatments on diverse biomasses, underscoring their potential for bioenergy production through bioconversion. Looking ahead, we will place more emphasis on developing novel pretreatment strategies of these classic, potential, emerging pretreatments and/or their combinations, hence providing a foundation for the application of biomass energy.

## Data Availability

The data and the materials are all available in this article.
